
JOURNAL INFORMATION
==============================
NLM Title Abbreviation: Rev Soc Bras Med Trop
Journal ID: Rev Soc Bras Med Trop
NLM Journal ID: 7507456
Journal ID: rsbmt

Revista da Sociedade Brasileira de Medicina Tropical

ISSN: 0037-8682
EISSN: 1678-9849
Publisher: Brazilian Society of Tropical Medicine

ARTICLE INFORMATION
==============================
PMCID: PMC10706049
PMID: 38088667
DOI: 10.1590/0037-8682-0549-2023
Article ID: 01001
Article version: 1
Subjects: Technical Report

Guidelines for Trypanosoma cruzi-HIV Co-infection and other Immunosuppressive Conditions: Diagnosis, Treatment, Monitoring, and Implementation from the International Network of Care and Studies - 2023

http://orcid.org/0000-0001-7619-9405 de Almeida Eros Antonio 1 Conception and design of the study Final approval of the version to be submitted Writing original draft Validation
http://orcid.org/0000-0003-2033-1715 Mendes Fernanda de Souza Nogueira Sardinha 2 Writing original draft Validation
http://orcid.org/0000-0001-7982-1757 Ramos Alberto Novaes Júnior 3 Conception and design of the study Writing original draft Validation
http://orcid.org/0000-0001-8266-4801 de Sousa Andréa Silvestre 2 Writing original draft Validation
http://orcid.org/0000-0001-5276-2099 Pavan Tycha Bianca Sabaini 4 Final approval of the version to be submitted Writing original draft Validation
http://orcid.org/0000-0001-6369-3631 Mediano Mauro Felippe Felix 2 Writing original draft Validation
http://orcid.org/0000-0003-0335-0877 Ostermayer Alejandro Luquetti 5 Writing original draft Validation
http://orcid.org/0000-0002-5430-7222 Hasslocher-Moreno Alejandro Marcel 2
http://orcid.org/0000-0003-0788-7030 Britto Constança Felicia De Paoli de Carvalho 6
http://orcid.org/0000-0002-1243-3060 Novaes Christina Gallafrio 7 Writing original draft Validation
http://orcid.org/0000-0002-2174-5058 Correia Dalmo 8 Writing original draft Validation
http://orcid.org/0000-0002-3944-0818 Santos Fred Luciano Neves 4 Writing original draft Validation
http://orcid.org/0000-0002-0468-4417 da Silva Gilberto Marcelo Sperandio 2 Writing original draft Validation
http://orcid.org/0000-0003-1789-4584 Fernandez Marisa Liliana 9 Writing original draft Validation
http://orcid.org/0000-0002-3012-8844 Lima Mayara Maia 10 Writing original draft
http://orcid.org/0000-0002-1884-9526 de Carvalho Noêmia Barbosa 7 Writing original draft Validation
http://orcid.org/0000-0002-5340-8697 Moreira Otacílio da Cruz 11
http://orcid.org/0000-0002-3530-8078 Albajar-Viñas Pedro 12 Writing original draft Validation
http://orcid.org/0000-0002-3244-6678 Leite Ruth Moreira 13 Writing original draft Validation
http://orcid.org/0000-0001-9389-2420 Palmeira Swamy Lima 10 Writing original draft
http://orcid.org/0000-0002-6829-8582 da Costa Veruska Maia 10 Writing original draft
http://orcid.org/0000-0003-3864-041X Yasuda Maria Aparecida Shikanai 7 Conception and design of the study Final approval of the version to be submitted Writing original draft Validation
1 Universidade Estadual de Campinas, Faculdade de Ciências Médicas, Grupo de Estudos em doença de Chagas, Campinas, SP, Brasil.
2 Fundação Oswaldo Cruz, Instituto Nacional de Infectologia Evandro Chagas, Rio de Janeiro, RJ, Brasil.
3 Universidade Federal do Ceará, Faculdade de Medicina, Programa de Pós-Graduação em Saúde Pública, Fortaleza, CE, Brasil.
4 Fundação Oswaldo Cruz, Instituto Gonçalo Moniz, Laboratório Avançado de Saúde Pública, Bahia, BA, Brasil.
5 Universidade Federal de Goiás, Hospital das Clínicas, Núcleo de Estudos da Doença de Chagas, Goiânia, GO, Brasil.
6 Fundação Oswaldo Cruz, Instituto Oswaldo Cruz, Laboratório de Biologia Molecular e Doenças Endêmicas, Rio de Janeiro, RJ, Brasil.
7 Universidade de São Paulo, Faculdade de Medicina, Departamento de Moléstias Infecciosas e Parasitárias, São Paulo, Brasil.
8 Universidade Federal de Sergipe, São Cristóvão, SE, Brasil.
9 Hospital de Infecciosas FJ Muñiz, Instituto Nacional de Parasitología “Dr. Mario Fatala Chabén”, Administración Nacional de Laboratorios e Institutos de Salud, Buenos Aires, Argentina.
10 Ministério da Saúde, Secretaria de Vigilância em Saúde, Brasília, DF, Brasil.
11 Fundação Oswaldo Cruz, Instituto Oswaldo Cruz, Laboratório de Virologia e Parasitologia Molecular, Rio de Janeiro, RJ, Brasil.
12 Department of Control of Neglected Tropical Diseases, World Health Organization, Geneva, Switzerland.
13 Centro de Vigilância Epidemiológica Professor Alexandre Vranjac. Secretaria de Estado da Saúde do estado de São Paulo, São Paulo, SP, Brasil.
Corresponding author: Eros Antonio de Almeida and Tycha Bianca Sabaini Pavan. e-mail: eros@unicamp.br; tychabianca@gmail.com Conflict of Interest: The authors declare that there is no conflict of interest.

Electronic publication date: 2023 Dec 8
Publication date: 2023
Volume: 56
Electronic Location ID: e0549-2023
Received 2023 Nov 10; Accepted 2023 Nov 16
License: Este é um artigo publicado em acesso aberto sob uma licença Creative Commons
License URL: https://creativecommons.org/licenses/by/4.0/

Figure count: 14
Table count: 1
Equation count: 0
Reference count: 101

==============================

REFERENCES

1 Dias JC Ramos AN Jr Gontijo ED Luquetti A Shikanai-Yasuda MA Coura JR 2nd Brazilian Consensus on Chagas Disease, 2015 Rev Soc Bras Med Trop 2016 49 Suppl 1 3 60 10.1590/0037-8682-0505-2016 27982292
2 Ramos AN. Jr Recomendações para diagnóstico, tratamento e acompanhamento da co-infecção Trypanosoma cruzi: vírus da imunodeficiência humana Rev Soc Bras Med Trop 2006 39 4 392 415 10.1590/S0037-86822006000400017 17119760
3 Shikanai-Yasuda MA Mediano MFF Novaes CTG Sousa AS Sartori AMC Santana RC Correction: Clinical profile and mortality in patients with T. cruzi/HIV co-infection from the multicenter data base of the "Network for healthcare and study of Trypanosoma cruzi/HIV co-infection and other immunosuppression conditions" PLoS Negl Trop Dis 2023 17 1 e0011036 10.1371/journal.pntd.0011036 Erratum for: PLoS Negl Trop Dis . 2021 Sep 30;15(9):e0009809 36598894 PMC9812302
4 Ministério da Saúde (MS). Comissão Nacional de Incorporação de Tecnologias no SUS (CONITEC) Protocolo Clínico e Diretrizes Terapêuticas Doença de Chagas Brasília MS 2018 135 p http://antigo-conitec.saude.gov.br/images/PCDT_Doenca_de_Chagas.pdf
5 Stanaway JD Roth G The burden of Chagas disease: estimates and challenges Glob Heart 2015 10 3 139 144 10.1016/j.gheart.2015.06.001 26407508
6 Shikanai Yasuda MA Emerging and reemerging forms of Trypanosoma cruzi transmission Mem Inst Oswaldo Cruz 2022 117 e210033 10.1590/0074-02760210033 35584508 PMC9113729
7 Chagas C Nova tripanozomiaze humana: estudos sobre a morfolojia e o ciclo evolutivo do Schizotrypanum cruzi n. gen., n. sp., ajente etiolojico de nova entidade morbida do homem Mem Inst Oswaldo Cruz 1909 1 2 159 218 10.1590/s0074-02761909000200008
8 World Health Organization (WHO) Chagas disease in Latin America: an epidemiological update based on 2010 estimates Wkly Epidemiol Rec 2015 90 6 33 43 25671846
9 Apt W Zulantay I Estado actual en el tratamiento de la enfermedad de Chagas [Update on the treatment of Chagas' disease] Rev Med Chil 2011 139 2 247 257 10.4067/S0034-98872011000200016 21773664
10 Sartori AM Ibrahim KY Nunes Westphalen EV Braz LM Oliveira OC Jr Gakiya E Manifestations of Chagas disease (American trypanosomiasis) in patients with HIV/AIDS Ann Trop Med Parasitol 2007 101 1 31 50 10.1179/136485907X154629 17244408
11 Acquatella H Echocardiography in Chagas heart disease Circulation 2007 115 9 1124 1131 10.1161/CIRCULATIONAHA.106.627323 17339570
12 Dias JC The indeterminate form of human chronic Chagas' disease A clinical epidemiological review Rev Soc Bras Med Trop 1989 22 3 147 156 10.1590/S0037-86821989000300007 2486527
13 Almeida EA Epidemiologia e clínica da coinfecção Trypanosoma cruzi e vírus da imunodeficiência adquirida 1st ed Campinas Unicamp 2015 328 p
14 Lattes R Linares L Radisic M Emerging parasitic infections in transplantation Curr Infect Dis Rep 2012 14 6 642 649 10.1007/s11908-012-0295-z 23054931
15 Cavalcanti MAF Neta FI Benevides DHJ Andrade CM Nascimento EGC Caracterizando o perfil de mortalidade da doença de Chagas no Brasil ANAIS MEDTROP 2016 https://www.sbmt.org.br/medtrop2016/wp-content/uploads/2016/10/10193-Caracterizando-o-perfil-de-mortalidade-da-doença-de-Chagas-no-Brasil.pdf
16 Pérez-Molina JA Molina I Chagas disease Lancet 2018 391 10115 82 94 10.1016/S0140-6736(17)31612-4 28673423
17 Pinto Dias JC Human chagas disease and migration in the context of globalization: some particular aspects J Trop Med 2013 2013 789758 789758 10.1155/2013/789758 23606862 PMC3625591
18 Ferreira I de LM Silva TPT e Eliminação da transmissão da doença de Chagas pelo Triatoma infestans no Brasil: um fato histórico Rev Soc Bras Med Trop 2006 39 5 507 509 10.1590/S0037-86822006000500018 17160334
19 World Health Organization (WHO) Chagas disease (American trypanosomiasis) - fact sheet (revised in August 2012) Wkly Epidemiol Rec 2012 87 51/52 519 522
20 Organización Panamericana de la Salud Guía para el diagnóstico y el tratamiento de la enfermedad de Chagas 2019 http://iris.paho.org/xmlui/handle/10665.2/49653
21 Freilij H Altcheh J Muchinik G Perinatal human immunodeficiency virus infection and congenital Chagas' disease Pediatr Infect Dis J 1995 14 2 161 162 7746707
22 Rassi A Jr Rassi A Marcondes de Rezende J American trypanosomiasis (Chagas disease) Infect Dis Clin North Am 2012 26 2 275 291 10.1016/j.idc.2012.03.002 22632639
23 Martins-Melo FR Ramos AN Jr Alencar CH Heukelbach J Mortality Related to Chagas Disease and HIV/AIDS Coinfection in Brazil J Trop Med 2012 2012 534649 534649 10.1155/2012/534649 22969814 PMC3434406
24 D'Albuquerque LA Gonzalez AM VN HL Filho Copstein JL Larrea FI Mansero JM Liver transplantation from deceased donors serologically positive for Chagas disease Am J Transplant 2007 7 3 680 684 10.1111/j.1600-6143.2007.01663.x 17217440
25 Organización Panamericana de la Salud Enfermedad de Chagas e inmunosupresión. Decálogo para la prevención, el diagnóstico y el tratamiento 2021 https://iris.paho.org/handle/10665.2/54561
26 Consolidated Guidelines on the Use of Antiretroviral Drugs for Treating and Preventing HIV Infection: Recommendations for a Public Health Approach 2nd ed Geneva World Health Organization 2016 https://www.who.int/publications/i/item/9789241549684 27466667
27 Almeida EA Ramos AN Júnior Correia D Shikanai-Yasuda MA Co-infection Trypanosoma cruzi/HIV: systematic review (1980-2010) Rev Soc Bras Med Trop 2011 44 6 762 770 10.1590/S0037-86822011000600021 22231251
28 Jannin J Presentation of the consensus workshop about the Chagas disease in non-endemic areas (26 June 2009, Paris, France) Bull Soc Pathol Exot 2009 102 5 275 275 20131418
29 Almeida EA Lima JN Lages-Silva E Guariento ME Aoki FH Torres-Morales AE Chagas' disease and HIV co-infection in patients without effective antiretroviral therapy: prevalence, clinical presentation and natural history Trans R Soc Trop Med Hyg 2010 104 7 447 452 10.1016/j.trstmh.2010.02.004 20303560
30 Cordova E Boschi A Ambrosioni J Cudos C Corti M Reactivation of Chagas disease with central nervous system involvement in HIV-infected patients in Argentina, 1992-2007 Int J Infect Dis 2008 12 6 587 592 10.1016/j.ijid.2007.12.007 18337139
31 Lopez-Albizu C Bravo MP Pico M Fernandez ML Case report of Chagas disease reactivation: new diagnosis tool by direct microscopic observation of biopsy specimen and its preservation fluid Rev Soc Bras Med Trop 2020 54 e20200326 10.1590/0037-8682-0326-2020 33338120 PMC7747806
32 Sartori AM E J Neto Nunes EV Braz LM Caiaffa-Filho HH Oliveira Oda C Jr Trypanosoma cruzi parasitemia in chronic Chagas disease: comparison between human immunodeficiency virus (HIV)-positive and HIV-negative patients J Infect Dis 2002 186 6 872 875 10.1086/342510 12198628
33 Benvenuti LA Roggério A Sambiase NV Fiorelli A Higuchi Mde L Polymerase chain reaction in endomyocardial biopsies for monitoring reactivation of Chagas' disease in heart transplantation: a case report and review of the literature Cardiovasc Pathol 2005 14 5 265 268 10.1016/j.carpath.2005.06.001 16168900
34 Benvenuti LA Roggério A Coelho G Fiorelli AI Usefulness of qualitative polymerase chain reaction for Trypanosoma cruzi DNA in endomyocardial biopsy specimens of chagasic heart transplant patients J Heart Lung Transplant 2011 30 7 799 804 10.1016/j.healun.2011.02.012 21481605
35 Burgos JM Diez M Vigliano C Bisio M Risso M Duffy T Molecular identification of Trypanosoma cruzi discrete typing units in end-stage chronic Chagas heart disease and reactivation after heart transplantation Clin Infect Dis 2010 51 5 485 495 10.1086/655680 20645859
36 Cuna WR Choque AG Passera R Rodriguez C Pro-inflammatory cytokine production in chagasic mothers and their uninfected newborns J Parasitol 2009 95 4 891 894 10.1645/GE-1927.1 19161249
37 Carlier Y Sosa-Estani S Luquetti AO Buekens P Congenital Chagas disease: an update Mem Inst Oswaldo Cruz 2015 110 3 363 368 10.1590/0074-02760140405 25760448 PMC4489473
38 Alonso-Vega C Hermann E Truyens C Rodriguez P Torrico MC Torrico F Estatus inmunológico de las madres infectadas por T. cruzi [Immunological status of mothers infected with Trypanosoma cruzi] Rev Soc Bras Med Trop 2005 38 Suppl 2 101 104 16482826
39 Scapellato PG Bottaro EG Rodríguez-Brieschke MT Mother-child transmission of Chagas disease: could coinfection with human immunodeficiency virus increase the risk? Rev Soc Bras Med Trop 2009 42 2 107 109 10.1590/S0037-86822009000200002 19448923
40 Bisio M Altcheh J Lattner J Moscatelli G Fink V Burgos JM Benznidazole treatment of chagasic encephalitis in pregnant woman with AIDS Emerg Infect Dis 2013 19 9 1490 1492 10.3201/eid1909.130667 23965334 PMC3810932
41 Castro CG de Jr Gregianin LJ Brunetto AL Transplante de medula óssea e transplante de sangue de cordão umbilical em pediatria J Pediatr Rio J 2001 77 5 345 360 10.1590/S0021-75572001000500004 14647838
42 Nisida IV Amato V Neto Braz LM Duarte MI Umezawa ES A survey of congenital Chagas' disease, carried out at three health institutions in São Paulo City, Brazil Rev Inst Med Trop Sao Paulo 1999 41 5 305 311 10.1590/S0036-46651999000500007 10602545
43 Vekemans J Truyens C Torrico F Solano M Torrico MC Rodriguez P Maternal Trypanosoma cruzi infection upregulates capacity of uninfected neonate cells To produce pro- and anti-inflammatory cytokines Infect Immun 2000 68 9 5430 5434 10.1128/IAI.68.9.5430-5434.2000 10948177 PMC101811
44 Agosti MR Ercoli P Dolcini G Andreani G Martínez Peralta L González Ayala S Two cases of mother-to-child transmission of HIV and Trypanosoma cruzi in Argentina Braz J Infect Dis 2012 16 4 398 399 10.1016/j.bjid.2012.06.008 22846134
45 Fernández ML Marson ME Mastrantonio GE Corti MA Fleitas U Lloveras SC Benznidazole in Cerebrospinal Fluid: a Case Series of Chagas Disease Meningoencephalitis in HIV-Positive Patients Antimicrob Agents Chemother 2021 65 3 e01922-20 10.1128/AAC.01922-20 33361290 PMC8092498
46 Portela-Lindoso AA Shikanai-Yasuda MA Doença de Chagas crônica: do xenodiagnóstico e hemocultura à reação em cadeia da polimerase [Chronic Chagas' disease: from xenodiagnosis and hemoculture to polymerase chain reaction]. Rev Saude Publica 2003 37 1 107 115 10.1590/s0034-89102003000100016 12488927
47 Ferreira MS Borges AS Some aspects of protozoan infections in immunocompromised patients- a review Mem Inst Oswaldo Cruz 2002 97 4 443 457 10.1590/S0074-02762002000400001 12118272
48 Duffy T Bisio M Altcheh J Burgos JM Diez M Levin MJ Accurate real-time PCR strategy for monitoring bloodstream parasitic loads in chagas disease patients PLoS Negl Trop Dis 2009 3 4 e419 10.1371/journal.pntd.0000419 19381287 PMC2667272
49 Cura CI Lattes R Nagel C Gimenez MJ Blanes M Calabuig E Early molecular diagnosis of acute Chagas disease after transplantation with organs from Trypanosoma cruzi-infected donors Am J Transplant 2013 13 12 3253 3261 10.1111/ajt.12487 24266974
50 Moreira OC Ramírez JD Velázquez E Melo MF Lima-Ferreira C Guhl F Towards the establishment of a consensus real-time qPCR to monitor Trypanosoma cruzi parasitemia in patients with chronic Chagas disease cardiomyopathy: a substudy from the BENEFIT trial Acta Trop 2013 125 1 23 31 10.1016/j.actatropica.2012.08.020 22982466
51 Martinez-Perez A Norman FF Monge-Maillo B Perez-Molina JA Lopez-Velez R An approach to the management of Trypanosoma cruzi infection (Chagas' disease) in immunocompromised patients Expert Rev Anti Infect Ther 2014 12 3 357 373 10.1586/14787210.2014.880652 24484076
52 Burgos JM Begher S Silva HM Bisio M Duffy T Levin MJ Molecular identification of Trypanosoma cruzi I tropism for central nervous system in Chagas reactivation due to AIDS Am J Trop Med Hyg 2008 78 2 294 297 18256432
53 Pacheco RS Ferreira MS Machado MI Brito CM Pires MQ Da-Cruz AM Chagas' disease and HIV co-infection: genotypic characterization of the Trypanosoma cruzi strain Mem Inst Oswaldo Cruz 1998 93 2 165 169 10.1590/S0074-02761998000200005 9698886
54 Burgos JM Begher SB Freitas JM Bisio M Duffy T Altcheh J Molecular diagnosis and typing of Trypanosoma cruzi populations and lineages in cerebral Chagas disease in a patient with AIDS Am J Trop Med Hyg 2005 73 6 1016 1018 16354804
55 Schijman AG Bisio M Orellana L Sued M Duffy T Mejia Jaramillo AM International study to evaluate PCR methods for detection of Trypanosoma cruzi DNA in blood samples from Chagas disease patients PLoS Negl Trop Dis 2011 5 1 e931 10.1371/journal.pntd.0000931 21264349 PMC3019106
56 Duffy T Cura CI Ramirez JC Abate T Cayo NM Parrado R Analytical performance of a multiplex Real-Time PCR assay using TaqMan probes for quantification of Trypanosoma cruzi satellite DNA in blood samples PLoS Negl Trop Dis 2013 7 1 e2000 10.1371/journal.pntd.0002000 23350002 PMC3547845
57 Andrade JP Marin JA Neto Paola AA Vilas-Boas F Oliveira GM Bacal F I Latin American Guidelines for the diagnosis and treatment of Chagas' heart disease: executive summary Arq Bras Cardiol 2011 96 6 434 442 10.1590/S0066-782X2011000600002 21789345
58 Marin-Neto JA Rassi A Jr Oliveira GMM Correia LCL Ramos AN Júnior Luquetti AO SBC Guideline on the Diagnosis and Treatment of Patients with Cardiomyopathy of Chagas Disease - 2023 Arq Bras Cardiol 2023 120 6 e20230269 10.36660/abc.20230269 37377258 PMC10344417
59 Vacas AS Gomez-Santana LV Torre AC Galimberti RL Reactivation of Chagas-Mazza disease during treatment with infliximab An Bras Dermatol 2017 92 6 899 900 10.1590/abd1806-4841.20177346 29364464
60 Rassi A Amato V Neto de Siqueira AF Doles J Leite MS Silva OQ Influência de corticóide, na doença de Chagas crônica, administrado em virtude de afecções associdas Rev Soc Bras Med Trop 1997 30 2 93 99 10.1590/S0037-86821997000200002 9148341
61 Lages-Silva E Ramirez LE Silva-Vergara ML Chiari E Chagasic meningoencephalitis in a patient with acquired immunodeficiency syndrome: diagnosis, follow-up, and genetic characterization of Trypanosoma cruzi Clin Infect Dis 2002 34 1 118 123 10.1086/324355 11731955
62 Pinazo MJ Espinosa G Cortes-Lletget C Posada Ede J Aldasoro E Oliveira I Immunosuppression and Chagas disease: a management challenge PLoS Negl Trop Dis 2013 7 1 e1965 10.1371/journal.pntd.0001965 23349998 PMC3547855
63 Molina I Gómez i Prat J Salvador F Treviño B Sulleiro E Serre N Randomized trial of posaconazole and benznidazole for chronic Chagas' disease N Engl J Med 2014 370 20 1899 1908 10.1056/NEJMoa1313122 24827034
64 Bacal F Silva CP Pires PV Mangini S Fiorelli AI Stolf NG Transplantation for Chagas' disease: an overview of immunosuppression and reactivation in the last two decades Clin Transplant 2010 24 2 E29-34 10.1111/j.1399-0012.2009.01202.x 20088914
65 Shikanai-Yasuda MA Novaes C G Freitas VLT Carvalho NB Experiência brasileira sobre a coinfecção T. cruzi/HIV Almeida EA Epidemiologia e clínica da coinfecção Trypanosoma cruzi e vírus da imunodeficiência adquirida 1st ed Campinas Unicamp 2015 298 308
66 Schijman AG Vigliano C Burgos J Favaloro R Perrone S Laguens R Early diagnosis of recurrence of Trypanosoma cruzi infection by polymerase chain reaction after heart transplantation of a chronic Chagas' heart disease patient J Heart Lung Transplant 2000 19 11 1114 1117 10.1016/S1053-2498(00)00168-6 11077230
67 Ministério da Saúde (MS). Secretaria de Vigilância em Saúde. Departamento de Doenças de Condições Crônicas e Infecções Sexualmente Transmissíveis Relatório de monitoramento clínico do HIV 2021 Brasília MS 2022 153 153 https://bvsms.saude.gov.br/bvs/publicacoes/relatorio_monitoramento_clinico_hiv_2021.pdf
68 Silva VLC Albuquerque LEJ Prevalência de infecção pelo T. cruzi em doadores de sangue nos hemocentros coordenadores do Brasil em 2007 Epidemiol. Serv. Saúde 2013 22 1 103 110 10.5123/S1679-49742013000100011
69 Kransdorf EP Zakowski PC Kobashigawa JA Chagas disease in solid organ and heart transplantation Curr Opin Infect Dis 2014 27 5 418 424 10.1097/QCO.0000000000000088 25023742
70 Salvador F Sánchez-Montalvá A Valerio L Serre N Roure S Treviño B Immunosuppression and Chagas disease; experience from a non-endemic country Clin Microbiol Infect 2015 21 9 854 860 10.1016/j.cmi.2015.05.033 26055418
71 Stauffert D Silveira MF Mesenburg MA Manta AB Dutra AD Bicca GL Prevalence of Trypanosoma cruzi/HIV coinfection in southern Brazil Braz J Infect Dis 2017 21 2 180 184 10.1016/j.bjid.2016.10.006 27914221 PMC9427564
72 McCormack L Quiñónez E Goldaracena N Anders M Rodríguez V Orozco Ganem F Liver transplantation using Chagas-infected donors in uninfected recipients: a single-center experience without prophylactic therapy Am J Transplant 2012 12 10 2832 2837 10.1111/j.1600-6143.2012.04160.x 22813351
73 Sousa AA Lobo MC Barbosa RA Bello V Chagas seropositive donors in kidney transplantation Transplant Proc 2004 36 4 868 869 10.1016/j.transproceed.2004.03.113 15194296
74 Lattes R Lasala MB Chagas disease in the immunosuppressed patient Clin Microbiol Infect 2014 20 4 300 309 10.1111/1469-0691.12585 24602129
75 Bern C Kjos S Yabsley MJ Montgomery SP Trypanosoma cruzi and Chagas' Disease in the United States Clin Microbiol Rev 2011 24 4 655 681 10.1128/CMR.00005-11 21976603 PMC3194829
76 Hermann E Truyens C Alonso-Vega C Rodriguez P Berthe A Torrico F Congenital transmission of Trypanosoma cruzi is associated with maternal enhanced parasitemia and decreased production of interferon- gamma in response to parasite antigens J Infect Dis 2004 189 7 1274 1281 10.1086/382511 15031797
77 Riarte A Luna C Sabatiello R Sinagra A Schiavelli R De Rissio A Chagas' disease in patients with kidney transplants: 7 years of experience 1989-1996 Clin Infect Dis 1999 29 3 561 567 10.1086/598634 10530448
78 Chagas' Disease Argentine Collaborative Transplant Consortium Casadei D Chagas' disease and solid organ transplantation Transplant Proc 2010 42 9 3354 3359 10.1016/j.transproceed.2010.09.019 21094779
79 Diez M Favaloro L Bertolotti A Burgos JM Vigliano C Lastra MP Usefulness of PCR strategies for early diagnosis of Chagas' disease reactivation and treatment follow-up in heart transplantation Am J Transplant 2007 7 6 1633 1640 10.1111/j.1600-6143.2007.01820.x 17511688
80 de Freitas VL da Silva SC Sartori AM Bezerra RC Westphalen EV Molina TD Real-time PCR in HIV/Trypanosoma cruzi coinfection with and without Chagas disease reactivation: association with HIV viral load and CD4 level PLoS Negl Trop Dis 2011 5 8 e1277 10.1371/journal.pntd.0001277 21912712 PMC3166046
81 Lescure FX Le Loup G Freilij H Develoux M Paris L Brutus L Chagas disease: changes in knowledge and management Lancet Infect Dis 2010 10 8 556 570 10.1016/S1473-3099(10)70098-0 20670903
82 Britto CC Usefulness of PCR-based assays to assess drug efficacy in Chagas disease chemotherapy: value and limitations Mem Inst Oswaldo Cruz 2009 104 Suppl 1 122 135 10.1590/S0074-02762009000900018 19753467
83 Campos SV Strabelli TM Amato V Neto Silva CP Bacal F Bocchi EA Risk factors for Chagas' disease reactivation after heart transplantation J Heart Lung Transplant 2008 27 6 597 602 10.1016/j.healun.2008.02.017 18503957
84 Dictar M Sinagra A Verón MT Luna C Dengra C De Rissio A Recipients and donors of bone marrow transplants suffering from Chagas' disease: management and preemptive therapy of parasitemia Bone Marrow Transplant 1998 21 4 391 393 10.1038/sj.bmt.1701107 9509974
85 Altclas J Sinagra A Dictar M Luna C Verón MT De Rissio AM Chagas disease in bone marrow transplantation: an approach to preemptive therapy Bone Marrow Transplant 2005 36 2 123 129 10.1038/sj.bmt.1705006 15908978
86 Sánchez-Montalvá A Salvador F Ruiz-Camps I Barba P Valcárcel D Sulleiro E Imported Disease Screening Prior to Chemotherapy and Bone Marrow Transplant ation for Oncohematological Malignancies Am J Trop Med Hyg 2016 95 6 1463 1468 10.4269/ajtmh.16-0458 27928093 PMC5154468
87 Huprikar S Bosserman E Patel G Moore A Pinney S Anyanwu A Donor-derived Trypanosoma cruzi infection in solid organ recipients in the United States, 2001-2011 Am J Transplant 2013 13 9 2418 2425 10.1111/ajt.12340 23837488 PMC11382615
88 Pinazo MJ Miranda B Rodríguez-Villar C Altclas J Brunet Serra M García-Otero EC Recommendations for management of Chagas disease in organ and hematopoietic tissue transplantation programs in nonendemic areas Transplant Rev Orlando 2011 25 3 91 101 10.1016/j.trre.2010.12.002 21530219
89 Coura JR de Abreu LL Willcox HP Petana W Comparative controlled study on the use of benznidazole, nifurtimox and placebo, in the chronic form of Chagas' disease, in a field area with interrupted transmission. I. Preliminary evaluation Rev Soc Bras Med Trop 1997 30 2 139 144 10.1590/S0037-86821997000200009 9148337
90 Dias JCP Coura JR Clínica e terapêutica da doença de Chagas: uma abordagem prática para o clínico geral 1 st. Ed Rio de Janeiro Editora Fiocruz 1997 486 p
91 Rassi A Amato V Neto de Siqueira AF Ferriolli F Filho Amato VS Rassi A Júnior Protective effect of benznidazole against parasite reactivation in patients chronically infected with Trypanosoma cruzi and treated with corticoids for associated diseases Rev Soc Bras Med Trop 1999 32 5 475 482 10.1590/S0037-86821999000500002 10881079
92 Bern C Chagas disease in the immunosuppressed host Curr Opin Infect Dis 2012 25 4 450 457 10.1097/QCO.0b013e328354f179 22614520
93 Lazo J Meneses AC Rocha A Ferreira MS Marquez JO Chapadeiro E Chagasic meningoencephalitis in the immunodeficient Arq Neuropsiquiatr 1998 56 1 93 97 10.1590/S0004-282X1998000100015 9686127
94 Benatti RD Oliveira GH Bacal F Heart Transplantation for Chagas Cardiomyopathy J Heart Lung Transplant 2017 36 6 597 603 10.1016/j.healun.2017.02.006 28284779
95 Reimer-McAtee MJ Mejia C Clark T Terle J Pajuelo MJ Cabeza J HIV and Chagas Disease: An Evaluation of the Use of Real-Time Quantitative Polymerase Chain Reaction to Measure Levels of Trypanosoma cruzi Parasitemia in HIV Patients in Cochabamba, Bolivia Am J Trop Med Hyg 2021 105 3 643 650 10.4269/ajtmh.20-1141 34398818 PMC8592353
96 Gray EB La Hoz RM Green JS Vikram HR Benedict T Rivera H Reactivation of Chagas disease among heart transplant recipients in the United States, 2012-2016 Transpl Infect Dis 2018 20 6 e12996 10.1111/tid.12996 30204269 PMC6289649
97 Salvador F Len O Molina I Sulleiro E Sauleda S Bilbao I Safety of liver transplantation with Chagas disease-seropositive donors for seronegative recipients Liver Transpl 2011 17 11 1304 1308 10.1002/lt.22346 21618698
98 dos Santos-Neto LL Polcheira MF Castro C Lima RA Simaan CK Corrêa-Lima FA Trypanosoma cruzi high parasitemia in patient with systemic lupus erythematosus Rev Soc Bras Med Trop 2003 36 5 613 615 14576877
99 Sánchez AG Baenas DF Bonisconti F Salinas MJH Alvarellos A Saurit V Reactivation of Chagas Disease in Patients With Rheumatic Autoimmune Diseases Diagnosed by Molecular Quantification Techniques J Clin Rheumatol 2021 27 8S S533 S536 10.1097/RHU.0000000000001108 31295157
100 Balouz V Agüero F Buscaglia CA Chagas Disease Diagnostic Applications: Present Knowledge and Future Steps Adv Parasitol 2017 97 1 45 10.1016/bs.apar.2016.10.001 28325368 PMC5363286
101 United Nations Programme on HIV/AIDS (UNAIDS) 90-90-90: uma meta ambiciosa de tratamento para contribuir para o fim da epidemia de Aids 2015 https://unaids.org.br/wp-content/uploads/2015/11/2015_11_20_UNAIDS_TRATAMENTO_META_PT_v4_GB.pdf
